# The experiences of female bisexual student-athletes in China: An interpretative phenomenological analysis

**DOI:** 10.3389/fpsyg.2023.1129961

**Published:** 2023-03-22

**Authors:** Meng Xiang, Kim Geok Soh, Yingying Xu, Seyedali Ahrari, Noor Syamilah Zakaria

**Affiliations:** ^1^Department of Sports Studies, Faculty of Educational Studies, Universiti Putra Malaysia, Seri Kembangan, Malaysia; ^2^Department of Philosophy and Civilization Studies, Faculty of Human Ecology, Universiti Putra Malaysia, Seri Kembangan, Malaysia; ^3^Department of Professional Development and Continuing Education, Faculty of Educational Studies, Universiti Putra Malaysia, Seri Kembangan, Malaysia; ^4^Department of Counsellor Education and Counselling Psychology, Faculty of Educational Studies, Universiti Putra Malaysia, Seri Kembangan, Malaysia

**Keywords:** bisexual, sport, student-athletes, China, sexual orientation

## Abstract

**Introduction:**

Many scholars have explored the participation of LGBTQ individuals in sports. However, these studies have either categorized homosexuality and bisexuality together or focused only on lesbian, gay, or transgender individuals. There is a lack of research in the literature on bisexual individuals’ sports participation and an even more significant lack of Asia perspectives. Therefore, this qualitative study is aimed to explore the experiences of female bisexual student-athletes in China.

**Methods:**

Semi-structured interviews with four female bisexual student-athletes were conducted and analyzed using Interpretative Phenomenological Analysis (IPA).

**Results:**

Three themes and eight sub-themes were identified. Theme 1. what bisexual identity means, and sub-themes: a struggling journey, emotional attraction, and gender role for female bisexual student-athletes; Theme 2. invalid identity, and sub-themes: lesbian mask, unrecognized identity; Theme 3. perceptions of sports context, and sub-themes: the influence of the sports context on sexual fluidity, relative inclusion, and perceived rejection.

**Conclusion:**

This study provides new insights into understanding the experience of female bisexual student-athlete. In addition, the results highlight the importance of the need to study bisexuality as a distinct identity.

## Introduction

Sport is a small society built on heterosexual masculinity. Hegemonic masculinity defined heterosexual men as the only legitimate identity, while women and sexual minority individuals were marginalized (Connell, 1995). Sports contributed to sexism, homophobia, and patriarchy (Anderson, 2009). With the development of society, Anderson found that hegemonic masculinity in sports was transitioning to inclusive masculinity, in which heterosexual male athletes inclusively accepted gay males. Although male athletes still wanted to prove heterosexuality, they did not discriminate against gay men and femininity (Anderson, 2009; McCormack, 2012). Despite the inclusive masculinity theory has been proven in several studies (Adams, 2011; Murray and White, 2017), Lesbian, gay, bisexual, transgender, and queer (LGBTQ) individuals currently still faced with prejudice and discrimination (Menzel et al., 2019; Knoester and Allison, 2021) and reported barriers to sport participation (Greenspan et al., 2019; Storr et al., 2021). Due to the conflicting results that emerged in the literature. Rollè et al. (2022) explained the complex attitudes of sports contexts toward LGBTQ individuals through a systematic review of 38 relevant studies. The results showed that among the younger generation, despite the continued adherence to orthodox masculinity and hidden discrimination, positive attitudes toward homosexuality were shown. Moreover, these positive attitudes need to be analyzed with caution, as a new form of discrimination against LGBTQ individuals may be carried out in a seemingly harmless way in the heterosexual and homonegative cultural system. However, while scholars have focused on the sports participation of LGBTQ individuals, issues related to bisexual individuals have been neglected. Previous studies either examined LGBTQ as a whole or focused only on lesbian, gay, or transgender individuals, and bisexual individuals-related studies were severely underrepresented in sports contexts (House et al., 2022). Only Anderson and Adams (2011) have studied male bisexuality in team sports. Scholar has called for more research that should focus on the experience of bisexuality as a distinct identity from lesbians and gay male (Barak, 2018).

According to the American Phycology of Association, Bisexuality is a sexual attraction or sexuality for both men and women. Bisexuality appears more common in women than men and varies across cultures (Vandenbos and American Psychological Association, 2015). As a distinct sexual identity, female bisexual individuals face significant mental health risks. Compared to lesbians and heterosexual women, bisexual women reported experiencing more peer appearance pressures (Hazzard et al., 2019) and worse mental health status, including anxiety, anger, depressive symptoms, self-injury, and suicidal ideation (Kerr et al., 2013). In addition, female bisexual individuals have often been devalued, and their bisexual identity was denied (Alarie and Gaudet, 2013; Woolley, 2020). Those negative experiences affected the healthy development of women’s identities.

As a country with a large population, China has a sizable LGBTQ community. In a national survey of Chinese college students released in 2020, non-heterosexuality accounted for 22.72% of the sample, with bisexual students accounting for 8.92% of the sample, making it the largest subgroup of the non-heterosexual sample (China Family Planning Association, 2020). However, despite the high percentage of bisexuality among youth, there has been no independent research on bisexuality-related issues in China. China is conservative when it comes to LGBTQ-related issues, both in society and in the sports context. The Winter Olympics in Beijing in 2022 featured a record number of openly LGBTQ athletes; however, none were from China (Lavietes, 2022). LGBTQ athletes in China also rarely come out publicly. So far, only one retired volleyball player has ever come out on social media; interestingly, the message was soon deleted (Buzinski, 2021). Therefore, Chinese LGBTQ athletes cannot express themselves freely.

Muñoz-Laboy (2019) suggested that conducting research in a specific racial/ethnic context allows for the examination of the intersectional dimensions of bisexuality, provides opportunities to explore unexamined areas of bisexuality, and offers the possibility of a social context for the bisexual expression. Hence the Chinese sports context may provide new insights into issues related to bisexuality. Therefore, as an intersectionality study of bisexuality, this study contributes to the literature by exploring the experiences of female bisexual student-athletes in China.

### Bisexuality and sexual fluidity of women

Bisexuality is a unique identity. It is neither straight nor gay/lesbian, nor is bisexuality a hybrid of, or middle ground between, the two (Voss et al., 2014). The term bisexuality is complex, and scholars have different views on what it means to be bisexual. Early studies defined bisexuality as an attraction to both men and women (Eisner, 2013). Swan (2018) described bisexuality as a collective term for a sexual orientation that includes sexual behavior or emotional attraction, emotional attachment, desire, or fantasy for both men and women. This definition emphasizes that attraction to bisexual individuals is not only based on gender but also on romantic and emotional affection. Another important definition is that bisexuality is not an equal preference for men and women. Early on, Weinberg et al. (1994) found five types of bisexuality: pure type, mid-type, heterosexual leaning, homosexual leaning, and varied. Only 2% of bisexual men and 17% of bisexual women showed a true equal preference for men and women (pure type). Thus, there were important group differences among bisexual individuals.

Both young women and men were found to have considerable sexual fluidity (Katz-Wise, 2015). Sexual fluidity is defined as the ability to depend on the flexibility of the situation in sexual response (Diamond, 2008). This flexibility allows individuals to experience changes in same-or opposite-sex desire at different times. Women’s sexual fluidity is different from that of men. Diamond et al. (2020) identified four types of sexual fluidity in women. Women’s sexuality is more likely to change than men’s and change over time and in different social contexts (Peplau and Garnets, 2000). Diamond (2003) found that over 5 years, more than a quarter of women gave up their lesbian/bisexual identity during that time: half reacquired a heterosexual identity, and a half gave up all identity labels. In addition, with sexual fluidity, females’ identities could become more complex because socialization teaches women to derive their identity from relationships with others and to seek sexuality in the context of emotional involvement, which could lead to greater variability in female identity as these relationships change (Rodríguez Rust, 2000).

### Sexual prejudice, biphobia, and mental health

Sexual prejudice is a particular form of homophobia and biphobia that occurs in sports contexts. Sexual prejudice refers to all negative attitudes based on sexual orientation and is always directed at those identified as gay males, lesbians, or bisexual individuals (Herek, 2000). Sexual prejudice was also evident in sports (Anderson and Mowatt, 2013; Piedra et al., 2017). In a national sports and society survey based on sexual prejudice, about 1 in 3 adults believed sports were not welcoming to LGBTQ athletes; nearly 40 percent reported experiencing sports-related abuse (Knoester and Allison, 2021). Baiocco et al. (2020) indicated three prejudice-related factors in exploring sexual prejudice on the sport scale: open rejection, denial of visibility, and gendered performance. These factors revealed traditional sexual prejudice, a belief that lesbian and gay athletes should be treated negatively because of their sexual orientation; a belief that sexual orientation is a private issue and should not be discussed; a belief that gay men are less competitive than heterosexuals, or a belief that lesbian women are not suited to play sports that are more suited to girls. Pistella et al. (2020) found that the experience of coming out in the family context could prevent the internalization of sexual prejudice, which may facilitate coming out in other life contexts. Meanwhile, studies have also found that sexual prejudice is closely related to cultural context (Piedra et al., 2017) and race (Walker and Melton, 2015). This leads to a belief that findings of sexual prejudice could vary by culture and race.

Despite as a subgroup of LGBTQ, unlike homophobia and transphobia, bisexual individuals experience a unique biphobia (Tavarez, 2022). Biphobia is prejudice, fear, or hatred directed at bisexual individuals. It takes the form of denial that bisexuality is a genuine sexual orientation or negative stereotypes of bisexual individuals. First, one form of bisexuality denial stemmed from a binary view of sexual orientation. An individual is either heterosexual or homosexual, with no position in between. Another denial is that bisexuality is an attempt rather than a natural sexual orientation (Yoshino, 2017). Denial of bisexuality can lead to bisexual erasure. Bisexual erasure refers to a lack of acknowledgment and ignoring the clear evidence that bisexuals exist, including not recognizing the category of “bisexual,” recognizing the category but excluding individuals from it, accepting an individual’s bisexuality as a stable identity but stigmatizing it (Yoshino, 2017, p. 203). In response to biphobia and bisexual erasure, bisexual individuals could develop internalized biphobia. People with internalized biphobia often hide, feel like impostors, or constantly question their sexuality. Swan (2018) identified bisexual stigma, cultural understanding of bisexuality, the desire to fit in with the heterosexual or homosexual community, and denial as reasons for bisexual individuals not identifying their bisexual identity. Additionally, studies showed that bisexual women are more likely to hide and not reveal their sexual identity for fear of rejection (Wandrey et al., 2015; Roberto et al., 2020).

For bisexual people, experiences of discrimination, bisexual invisibility/erasure, and lack of affirmative support for bisexuality have become hidden traumas that affect their physical and mental health (Ross et al., 2018; Arnett Iii et al., 2019). For instance, bisexual people suffered significantly higher rates of depression and anxiety (Ross et al., 2018) and poverty than lesbians and gay men (Badgett, 2018). Furthermore, increased anxiety and depression and response to negative life events may lead bisexual individuals to have a higher risk of non-suicidal self-injury (NSSI) than heterosexual individuals, gay men, and lesbian individuals (Dunlop et al., 2020). In addition, studies found that bisexual women showed a higher likelihood of frequent mental distress and overall poor health than lesbians (Fredriksen-Goldsen et al., 2010) and were at significantly greater risk for multiple substance use behaviors relative to lesbian (Schuler and Collins, 2020).

### Sexual minority females in sports participation

Research on bisexuality-related issues in sports is particularly limited; only a few studies have been conducted in the last decade. Anderson was one of the first scholars to explore the issue of bisexuality in sports. Anderson and Adams (2011) interviewed 60 male soccer players at three U.S. universities and found that these athletes considered bisexuality a legitimate, non-discriminatory sexual identity. Nevertheless, female bisexual student-athletes’ experiences in sports context may be different.

First, finding female bisexual participants in sports is difficult because the sexual orientation of female sexual minorities is fluid and continuous. The most common terms used for sexual identity in studies of female sexual minority individuals in the sports context are “lesbian,” “gay,” “bisexual,” and “queer.” In some studies, lesbian student-athletes used “gay” instead of” lesbian” (Stoelting, 2011; Melton and Cunningham, 2012; Mann and Krane, 2018). It seems female minority student-athletes preferred the fluidity of “gay” and tended to identify more with the continuum regarding sexual orientation (Fynes and Fisher, 2016). In a case, Ravel and Rail (2008) interviewed 14 Canadian female athletes who self-identified as lesbian, bisexual, and queer, observing that sexuality was often constructed as fluid, ever-changing, and resistant to the heterosexual/queer dichotomy. For these women, casual constructions of sexuality undermine notions of fixed identity while downplaying the “once and for all” process of coming out.

Secondly, women’s same-sex attraction may be related to the sports context. Davis-Delano (2014) interviewed 56 women who had at least one same-sex relationship by age 30 and found that sports nurtured and hindered the development of these relationships. If homophobia is prevalent on the team, it hinders the development of same-sex relationships for females. Conversely, sports fostered female same-sex relationships because women could demonstrate their abilities in sports, and there was a lack of a heteronormative atmosphere in women’s teams. Although these participants did not self-identify as lesbian or bisexual, this study revealed a subtle relationship between women’s same-sex attraction and the sports context.

Thirdly, the sports context may be more pressure on female sexual minorities than on gay males. Anderson and Bullingham (2015) summarized former studies of female sexual minority athletes and argued that women’s sports teams appear more hostile to sexual minorities than men’s teams. When a female athlete came out in a team sport, cultural suspicion of homosexuality was likely to be projected onto other female athletes because there were many lesbians in sports. The fear of becoming lesbian can fill the team with a homophobic climate. This is consistent with earlier research that female athletes with masculinity were labeled as lesbian, thus denying women’s success and dividing women’s teams (Krane, 1997).

### Minority stress theory

Minority stress theory (2003) was often applied to LGBTQ-related research. Several studies have adapted the minority stress theory as a theoretical perspective to examine the experiences of LGBTQ athletes in the sports context (Symons et al., 2017; Hartmann-Tews et al., 2020). Meyer (2003) found that stress might impact the health of LGB people through distal to proximal stress processes. Distal stress processes are prejudicial events, discrimination, and violence from the outside world directed toward LGB individuals. Expectations of rejection, concealment and internalized homophobia are proximal stress processes; they are the LGB individual’s experiences of discrimination and violence. Most importantly, this theory also explains the process of minority identity development and how minorities shape their identities in these experiences. Past research has shown the applicability of the theory in bisexuality-related research (Arnett Iii et al., 2019).

### Current study

China’s attitude toward LGBTQ-related issues determined the lag of its subgroup research. China removed homosexuality from mental disorders in 2003 (Chinese Classification of Mental Disorders, 2003). However, the Chinese government remained silent on LGBT issues, which has led to various forms of discrimination and prejudice against LGBTQ people. More than 18,000 Chinese LGBTQ people responded to a United Nations survey of LGBTQ-related issues in China. Half reported experiencing discrimination or abuse in school, the workplace, and, most often, within their own families (United Nations Development Programme, 2016). In another national survey, Wang et al. (2020) also found that Chinese LGBTQ people reported particularly high levels of discrimination from family and society. In China, the focus on LGBTQ-related issues is still on HIV prevention of gay populations, while lesbian and bisexual females individuals are neglected. One Asian scholar indicated that, in Asia, the patriarchal context, coupled with heterosexism, has led to ignorance of female lesbian/bisexual activity in countries like China (Ayutthaya, n.d.).

Therefore, given the prejudice against women and sexual minorities of the prevalence of heteronormative culture in sports contexts, the unique identity and complex situation of bisexual females, and the lack of Asian perspectives in the literature. This study aimed to explore the phenomenon of female‘bisexual identity from the perspectival experience of female bisexual student-athletes in the Chinese sports context. The research questions for this study were:

1. What are the perceptions of female bisexual student-athletes within the sports context regarding bisexual identity?
2. What are the experiences of being a female bisexual student-athlete in China?

## Materials and methods

### Study design

This study is a case study of an ongoing, university-funded qualitative research program focused on sexual minority student-athletes’ experiences in China. In this study, we specifically focus on the experience of female bisexual student-athletes in the sports context.

Interpretative Phenomenological Analysis (IPA) was adopted to explore the experiences of female bisexual student-athletes in the sports context in China. IPA is a qualitative approach based on phenomenology, hermeneutics, and idiographic IPA Focus on personal meaning and sensemaking for people who share a particular experience in a particular context (Smith et al., 2021). In addition, IPA lends itself to studies focusing on identity and a sense of self (Eatough and Smith, 2017). Therefore, IPA was considered an appropriate method to explore female bisexual student-athletes’ perceptions and experiences regarding bisexual identity in sports participation. Furthermore, The COREQ checklist was used for the report of this study (Tong et al., 2007).

### Research team and reflexivity

Reflexivity is vital in qualitative research and is an ongoing process that continues throughout the research effort (Olmos-Vega et al., 2022). This study emphasized reflexivity throughout the process, and the first author also presented personal reflexivity with the guidance of Clarke and Braun (2021).

The research team consisted of four women and one man. The researchers worked as Ph.D. students and professors in the Faculty of Education Studies and were all trained in qualitative research. The first author (MX) is a Chinese female Ph.D. student whose major is physical education. MX has received systematic qualitative training and is proficient in qualitative analysis software (NVivo). MX is a former student-athlete involved in long-term varsity competitions and currently working as a coach. Lesbians were common on women’s teams, and the college sports context seemed to provide an inclusive and safe space for sexual minority women. This phenomenon has attracted the interest of MX. Although MX was a student-athlete before, she tried to avoid the influence her experience had on this study. In addition, Participants were informed of the first author’s work background and the study’s objectives during the recruitment.

### Participant and procedure

Smith and Nizza (2022) recommended using purposive sampling in IPA studies to facilitate the recruitment of a homogeneous sample; they also suggested that snowballing can be an effective recruitment method when target participants are difficult to recruit. Thus, purposive sampling ad snowball sampling (Merriam and Tisdell, 2015) were used in this study. The study selected participants regarding Swan (2018)’s definition of bisexuality in terms of self-identity, behavior, and emotional attachment. Recruited female bisexual student-athletes had to meet the following criteria: (1) female; (2) self-identify as bisexual or be sexually or emotionally attracted to both males and females, and (3) be involved in intercollegiate sports or have participated in intercollegiate sports (no more than 3 years).

After receiving approval from the University’s Human Subjects Review Board, we asked lesbian student-athletes we knew to contact potential participants. First, potential participants were informed of the purpose and process of the study. Then, if the potential participant is interested and agrees, the contact person will give us the potential participant’s contact information. Then, we would decide whether to recruit and schedule an interview. We contacted nine potential participants, five of whom were rejected due to fear of disclosure. In the end, four female participants were recruited. Table 1 shows the pseudonyms, age, sports, sports status, years of participation in sports, sexual orientation, and interview information.

**Table 1:** Demographic characteristics of the participants.

Pseudonym	Age	Current/Former	Sport	Years of participation in sports	Sexual orientation	Number of interviews	Interview method
Joy	24	Current	Volleyball and Basketball	Six years	Bisexual	2	Face to face
Nicole	25	Former	Volleyball	Six years	Bisexual	2	Face to face
Kinsley	24	Current	Volleyball and Soccer	Five years	Bisexual	1	Face to face
Zoe	23	Current	Volleyball	Five years	Bisexual	1	Social software

Notably, in interpretive phenomenological research, saturation is usually not the objective, as the research is more concerned with obtaining a rich personal experience (van Manen et al., 2016). In IPA research, the sample size is not the determining factor. IPA research was conducted in a relatively small homogeneous sample to allow richness and depth of description in the analysis process (Smith and Nizza, 2022).

Due to the sensitivity of the LGBTQ-related topic in China, we used pseudonyms and omitted the names of the universities where the student-athletes competed to ensure their anonymity. This study fully accounted for the participants’ sensitive sexual identity in Chinese society. Before the study began, we contacted the participants and informed them of the general purpose of the study. We also assured participants were voluntary. During the data collection process, we avoided leading questions and withheld sharing personal impressions. In addition, to protect the participants’ privacy, the recorded interviews, photos, and videos were deleted once transcribed. The first author kept the participant’s transcripts on one passworded computer.

According to the scholars’ recommendation for IPA research (Smith et al., 2021; Smith and Nizza, 2022), an interview guide (Supplementary Appendix 1) was designed to help with data collection. The interview questions were drawn from the relevant literature and the researchers’ personal experiences. The interview guide centered on open-ended questions about participants’ thoughts and feelings about bisexuality, perception of bisexual identity, and experiences regarding bisexual identity. Before the official interview, we interviewed colleagues to ensure the interview questions were straightforward and easy to answer, changing some sensitive words at this stage. Then, a short pilot interview was conducted with one participant; minor modifications were made to the interview guide. Two authors (MX & YX) conducted the interviews. MX hosted the interview, and YX supervised the recording equipment. Before the interview, participants signed the informed consent form. Then, the semi-structured interviews began. Participants chose how, when, and where to conduct the interviews. Interviews included face-to-face and WeChat (China social software) voice chat interviews. The face-to-face interview locations were parks and cafes. Each interview lasted between 40 and 90 min and was audio recorded using two cell phones. Two participants were interviewed twice because one participant provided interesting information, and we wanted to go deeper with the second interview; the other participant conducted a pilot and a formal interview. Although we used an interview guide, the purpose of the interview guide in the IPA study is to only provide the researcher with a model (Smith and Nizza, 2022). So, we kept an open mind, flexibility, and focus during the interviews, striving to immerse ourselves in another person’s world, aiming to see the participants’ experiences through their eyes and thus understand how they make sense of the experiences of being female bisexual student-athletes.

In addition to the semi-structured interviews, we also collected data from documents (Creswell and Poth, 2016), which were derived from photos and videos from social media related to the topic of the study that the participants agreed to use, as well as the text of a participant’s chat content with a psychologist. Memos (Merriam and Tisdell, 2015) are another essential data source in qualitative research. We recorded what we heard, saw, and experienced during the collection and reflection process. These additional sources can be helpful in the subsequent contextualization of the data analysis (Smith et al., 2021) and provide the basis for the audit trail.

### Data analysis

The data analysis process was conducted by the seven steps of the IPA study guidelines and used the latest term of the analysis process of the IPA study (Smith et al., 2021). Two authors (MX and YX) conducted the data analysis. The first author used NVivo (QSR International Pty Ltd, 2020) to do the coding process, and the other used manual coding.

The first two steps were repeated reading and exploratory noting. After the interview recordings were translated into transcripts by the audio software, the first author (MX) listened to the recordings to correct the transcripts, and another author (YX) listened to the recording again and ensured the recordings were transcribed verbatim. Then, the transcripts were read repeatedly. Repeated readings ensured that the participants were the focus of the analysis and allowed for modeling the overall interview structure (Smith et al., 2021). While reading, some phrases or sentences were coded into exploratory notes. At this stage, we strove to keep the exploratory notes (codes) specific and visible. The next three steps were to construct the exploratory notes into experiential statements, cluster and compile the experiential statements, and name individual experiential themes. We kept an open mind in the process. We strove to explore and innovate so that the final experiential statements be structured together to produce a structure representing the most critical part of the participants’ description. An example of personal empirical themes is shown in Table 2. In the last two steps, we continued the same analysis for the other cases and developed the cross-case group experiential themes. All conflicts of opinion were resolved through discussions.

**Table 2:** Personal experiential themes for Zoe.

Quotes	
Theme 1. Perceptions of bisexuality	
Reflections on bisexual identity	*“I’ve spent a lot of time thinking about this.”*
Bisexuality is a heavy identity	*“This is a relatively heavy thing.”*
The intertwining of love and friendship	*“It’s possible for two feelings to be intertwined.”*
Confused about bisexual identity.	*“I felt a little confused; I felt that I did not know whether to date a boyfriend or a girlfriend.”*
The quest for security	*“This kind of relationship gives me emotional support and gives me a sense of security.”*
Heterosexual norms	*“I only like women who are a little more masculine in appearance.”*
One of the characteristics of being bisexual is having multiple choices	*“If I can like women and fall in love with men, then why do not I choose the easy way?”*
An intimate relationship with teammates	*“It’s great to have a close lover who can enjoy volleyball with you.”*
Theme 2. Bisexual as an invalid identity	
Bisexuality is perceived as being uncommitted.	*“She thinks my feelings are not strong, and I know it’s fruitless.”*
Either heterosexual or homosexual	*“Sexual orientation is a binary division, and there is no middle identity.”*
Unimportant identities	*“They may think you are joking, or you do not think clearly; it is not very important.”*
Bisexual is perceived as a not real identity	*“You may not really like women.”*
Bisexuality is considered just for fun	*“I just do that for fun.”*
Lack of understanding of bisexuality	*“Unfortunately, few people understand what bisexuality is.”*
Theme 3. Negative experiences	
Warned by coach	*“She (coach) told me that she would not allow this on her team.”*
Cursed by an ex-boyfriend (male student-athlete) regarding her bisexual identity	*“My ex-boyfriend (male student-athlete) was devastated when he found out that I liked girls, and then he cursed me on social media platforms.”*
Leaving the team due to relationship problems	*“My ex-boyfriend instigated my teammates to ostracize me, and I could not train anymore, so I left for a long time.”*
Unacceptable bisexual identity in the sports context	*“And people might think what you are doing, and it’s not acceptable to behave like that.”*
Theme 4. Negotiating identity	
Constantly trying to reveal her bisexual identity.	*“When I tried to mention to some friends that.”*
Want to share with good teammates	*“Because we were close teammates, and then I felt that since I had a new relationship, I wanted to be able to share it with them.”*
Selective disclosure	*“I will talk to my friends and teammates who are close to me, but I will not tell anyone else.”*
Wandering as a bisexual female	*“I would be so wandering, I like women, but I do not seem to be able to be with women all the time.”*
I just want to be a normal person.	*“In the future, I will choose to become like a normal woman. It’s easier this way.”*
Theme 5. Inclusion in the sports context	
The new generation is more inclusive.	*“The younger generation is receptive (for sexual minority females).”*
Lesbians are common in sports.	*“I know some friends who are on the soccer team, and they say that this phenomenon (lesbian) is quite common.”*
It provides more opportunities for contact with women	*“Sports teams have this kind of opportunity to be all women, without the contact of men. It will give women the possibility to explore more.”*
A close partner on the court	*“It’s great to have a close lover who can enjoy volleyball with you.”*
More accepting of female sexual minority students than gay men	*“Gay to be very scary.”; “It seems that lesbians are fresher now so that the acceptance may be higher.”*

To ensure rigor, this study complied with the IPA quality evaluation guide (Smith, 2011), including an explicit subscription to the IPA’s theoretical principles, adequate sampling from the corpus to show the density of evidence for each theme, and an in-depth analysis of the topics. In addition, this study also employed member checking (Merriam and Tisdell, 2015) and independent audits (Smith et al., 2021). The transcripts and analysis reports were sent back to the participant to obtain additional information and correct it; this allowed for the emergence of a co-constructed meaning from participants and researchers. Moreover, we documented the research steps taken throughout the study, including the initial notes on the research question, the interview guide, recorded transcripts, annotated to trace the process of establishing the analysis, and the researcher’s reflectivity, to make the study process transparent, enhancing trustworthiness in qualitative Inquiry (Carcary, 2009; Shinebourne, 2011).

## Results

This study aimed to explore the experiences of female bisexual student-athletes in the sports context in China. Specifically, to explore their perceptions of bisexual identity and their experiences as female bisexual student-athletes. Findings revealed three themes (1) what bisexual identity means, (2) invalid identity, (3) perceptions of sports context. The main themes, corresponding subthemes, and occurrence are shown in Table 3.

**Table 3:** Group experiential themes and subthemes.

Themes	Subthemes	Occurrence
What bisexual identity means	A struggling journey	Joy, Nicole, Kinsley, Zoe
	Emotional attraction	Joy, Nicole, Kinsley, Zoe
	Gender role for female bisexual student-athletes	Joy, Nicole, Kinsley, Zoe
Invalid identity	Lesbian mask	Joy, Nicole, Kinsley
	Unrecognized identity	Joy, Kinsley, Zoe
Perceptions of the sports context	The influence of the sports context on sexual fluidity	Joy, Nicole, Zoe
	Relative inclusion	Joy, Nicole, Kinsley, Zoe
	Perceived rejection	Joy, Nicole, Kinsley, Zoe

### What bisexual identity means

This theme refers to what bisexual identity means to female bisexual student-athletes, how they understand the concept of bisexuality, and how they view bisexual identity in the sports context. All participants described the struggles in perceiving their bisexual identity in the early stage. Participants perceived bisexuality as an identity of emotional attraction over gender. In addition, participants felt that they still needed to abide by heterosexual norms in the sports context.

### A struggling journey

Struggle represents the range of feelings that female bisexual student-athletes experience when confronted with their bisexual identity, including confusion, chaos, and worry. Zoe felt that facing her bisexual identity was confusing:

“I think there is a process, that is, at the beginning of that time, right before the age of 20, I felt a little confused; I felt that I did not know whether to date a boyfriend or a girlfriend; I don't know how others see me either […] no one can help me […] I was thinking about it all the time, I've spent a lot of time thinking about this” (Zoe).

Bisexual identity seemed strange to the participants, leaving them to wonder what was happening to them. For example, Nicole used the word “chaos” when asked about her initial confrontation with her bisexuality. “I had not been exposed to these concepts (bisexuality) but had developed these feelings and was quite confused, just like chaos” (Nicole). Similarly, Joy thought it was hard to understand this definition of bisexuality:

“At first, I thought I was heterosexual, then I thought I was a lesbian, and when I started dating men again, I couldn't find myself; it was like I didn't know what I was doing […] I would question what I was doing, ‘what the hell am I doing … Why do I make it so complicated’” (Joy).

Other participants described bisexuality as a wrong identity. Kinsley said, “I thought bisexual identity was wrong from the start because I do not think people should do that” (Kinsley). Thus, for the participants, understanding bisexual identity seemed to present a significant challenge. The process of coming to know one’s identity was difficult and lonely.

In addition, negative feelings about bisexual identity can have serious mental health implications. Joy thought that constantly thinking about her sexual identity had caused her great stress. Joy mentioned, “I suffered from an eating disorder since college, and I cannot say it was all because of my sexual orientation, but it was a big part of it” (Joy). She described that time as the most troubled time of her life:

“I don't know why; I just can't eat or make it hard to eat, and then felt incredibly guilty. I didn't want to think; it seemed like eating all the time would stop me from thinking, […] It was quite a long time, and I felt at that time that I might not live to be 25” (Joy).

While experiencing an eating disorder, Joy was diagnosed with depression:

“I couldn't sleep all night and didn't want to be too lonely; then I turned on the music. Then, after a while, I started crying for no reason […] those medications from the doctor didn't help me at all […] I can’t tell anyone. It's a shame to be like that” (Joy).

Joy used “guilty” and “shame” to describe her feelings, indicating Joy could not accept being a bisexual woman. “Guilty” and “shame” is a strong sense of self, yet always concerned with the gaze of others. It is irrefutable internal testimony that Joy was not living up to the standards (heterosexual women) she thought she should and feared that others would discover her bisexual identity. Thus, these fears created severe mental health risks for Joy.

### Emotional attraction

Participants agreed that emotional attraction was the primary prerequisite for entering a relationship. For example, Kinsley said, “I think it’s the emotional attraction that makes me accept women […] I do not want to think about gender” (Kinsley). Similarly, Nicole stated, “I did not know what bisexual was at the first time, but I knew I cared about that person, that intimate relationship, and that was the most important thing to me” (Nicole). Joy also indicated that the most immediate attraction to her was emotional:

“I knew I liked men because the attraction was obvious, but I knew the feeling was the same when I met my first girlfriend. Although I have to admit others may have influenced me (teammates) […] anyway, I really didn't have that hesitation at all, and I knew I liked her more than the feelings of a teammate” (Joy).

Kinsley believed that emotional attraction with women is more profound than a heterosexual relationship: “You get to train and compete with people you love, and there is no substitute for that kind of bond and trust” (Kinsley). The participants all had the experience of being in love with their teammates, and they trusted this relationship of being both lovers and friends. Zoe described this intertwined emotion of love and friendship with a teammate:

“I think it should be loved first and then friendship. The attraction of love is the first step, and then the mode of the relationship may have the friendship part […] If I am with a woman I like, I want to have a friendship with her, and I want to have love with her at the same time. It's possible for two feelings to be intertwined. It may not be the same as men” (Zoe).

For female bisexual student-athletes, the emotional attraction seems to be the primary prerequisite for entering a relationship. At the same time, participants all described intimate emotions with women that seemed to be warm and trusting.

### Gender role for female bisexual student-athletes

Despite being explicit about their bisexual identity, some participants reported having to conform to heterosexual gender roles in the sports context. Like many lesbians, female bisexual student-athletes either chose to be a “T” or a “P.” Those terms stand for the prevalent lesbian gender roles of butch and femme, especially stand for the tomboy and tomboy’s wife in China (UNDP and USAID, 2014). Femme refers to non-heterosexual women who behave and look feminine, while masculine non-heterosexual women are referred to as butch (Luoto et al., 2019b).

As the team captain, Nicole considered her role as, first and foremost, that of a “protector.” She believed the character should “remain tough and not allow weakness.” At the same time, most of her teammates considered her to be a “T”:

“I think they (teammates) all think I'm a lesbian, right […] If you were to ask me about my role as lesbian, it would definitely be T because of my appearance and my personality, […] I have short hair. I keep my hair short on the team because it's convenient for training […] I wouldn’t say I like to dress too femininely because I don't care much about appearance. Nevertheless, most of the time, people will define your role based on your appearance” (Nicole).

Unlike Nicole, the other three participants felt they were often treated as a “P” because of their feminine appearance. Some participants felt this male and female role between women was acceptable:

“Whether I fall in love with a woman or a man, I still want my other half to be like a male role […] the important thing is whether the role he/she plays in our relationship can give me a sense of security” (Kinsley).

This description appeared to be a complex need, as participants indicated that they did not care about gender; however, they felt that sense of security and male appearance were linked. “I only like women who are a little more masculine in appearance […], and this kind of relationship gives me emotional support and gives me a sense of security” (Zoe).

Notably, the protector role described by Nicole and the security needed by Kingsley and Zoe was related to the male role in society. It appeared that heterosexual norms were deeply entrenched in sexual minorities and subconsciously influenced the intimate relationship patterns of female sexual minorities.

Joy described her incomprehension with this phenomenon: “This is a bit unacceptable to me; we are both women; why should our pattern of loving imitate heterosexuality? It destroys the essence of our identities” (Joy). When asked why participants obey these binary heterosexual norms, Nicole argued that “this perception is a result of culture, and that Chinese culture lacks education on diversity, and that people only know the male and female roles, even if they are lesbian […] you have no way but to accept that” (Nicole). Nicole also expressed her dissatisfaction with the phenomenon:

“Being treated like a ‘T’ sometimes makes me feel uncomfortable. I'm also a woman, and I don't want to be treated like a male role in a relationship, like, to protect the patterner and so on; I think it should be mutual […] you would doubt whether the partner likes me or a male” (Nicole).

### Invalid identity

According to the participants’ descriptions, bisexual identity is an invalid identity. One’s sexual orientation is either homosexual or heterosexual; no matter how hard the participants try, bisexual identity is unimportant and unrecognized.

### Lesbian mask

The invalidation of bisexual identity is first demonstrated by the fact that female bisexuals were often treated as lesbians in the team. Joy found that she was the only bisexual student-athlete on the team, and the other teammates considered themselves lesbian or heterosexual; she found no place for bisexuals:

“What I can't fit in is that the team is in the lesbian camp on one side and the heterosexual camp on the other […], and I'm just embarrassed that I have to be a standard lesbian if I want to continue to try to date women” (Joy).

“[…] if I want to fit in (the team), I have to accept the lesbian identity because, for many people, the sexual orientation is not as complicated as ours […] You know, if you say you're a bisexual woman, you'll be ostracized by them (teammates) […] people don't like this (bisexuality) topic; the best way is to be silent; what they think is what it is” (Nicole).

Joy added, “People do not like bisexual women that much. A heterosexual teammate told me that ‘it’s not good to try everything.’ So, being treated as a lesbian is the easiest way” (Joy). Similarly, Kinsley said, “female bisexual student-athlete is often be considered as ‘easy’ woman, so if I’m perceived as a lesbian, it’s the safest and most comfortable solution to simply be a lesbian” (Kinsley). These descriptions expressed the participants’ tolerance and dissatisfaction with lesbian identity. On the one hand, female bisexual student-athletes wanted to fit in with the team; on the other hand, fitting in as lesbians made them hide their bisexual identity.

### Unrecognized identity

Some participants described their experiences of being rejected by the team for attempts to come out as bisexual:

“When I try to tell some of my teammates that I may have an intimate relationship with a woman before. People's reaction is first surprised, and then they will say, ‘you may not really like women’ or something like that. They may think you are joking, or you don’t think clearly; it is not very important. They would think […] that I just do that for fun” (Zoe).

To Zoe’s description, when she tried to come out to her teammates, her bisexual identity was “not real,” “not think clearly,” “unimportant,” or “for fun.” These terms proved that in the sports context, People believed that bisexual identity was unimportant and unrecognized. “People think that if you fall in love with a woman, you are a lesbian; if you fall in love with a man, you are a heterosexual; sexual orientation is a binary division, and there is no middle identity” (Zoe). “It’s like when people think you are a straight person who becomes a lesbian, and then because of something, you become straight again; you cannot be both ways” (Joy).

Not only do the teammates not recognize bisexuality, but some participants also believed that bisexual identity did not last long:

“I think it could all just be a college experience […] I've known some (bisexual student-athletes) before who have broken up and just graduated, and it was over. Most of them return to their normal (heterosexual) lives […] I don't think I can struggle with that; I'll probability marry a man” (Kinsley).

Kingsley used “normal” to describe heterosexuality. When we assume something is normal, anything beyond that boundary becomes abnormal. Even though Kingsley accepted her bisexual identity, it may be that in Kingsley's subconscious, bisexuality is not a normal identity. Similarly, Zoe assumed that “if I can like women and fall in love with men, then why don't I choose the easy way?” (Zoe).

### Perceptions of the sports context

This theme represents the perceptions of female bisexual student-athletes regarding the sports context. All participants agreed that the sports context influenced their sexuality to some extent. In addition, the sports context was relatively inclusive compared to other contexts in China. However, some participants reported negative experiences based on bisexual identity.

### The influence of the sporting context on sexual fluidity

Participants all indicated that there was a link between their sexuality and the sports context. First, participants reported that lesbian was common on sports teams, making them feel safe to fall into relationships with women in a sports context. “At that time, half of my teammates were lesbians, and some were dating soccer players” (Joy).

“Sports teams are full of masculine women […] I was on the volleyball team, and there were about 3/10 (lesbians) of the team […] I usually participate in some other sports, and in the case of our university, the soccer and basketball teams are basically lesbian couples […] (lesbians) are more than half. There are more lesbians on the soccer and basketball teams than on the volleyball team” (Kingsley).

Joy admitted she started to be attracted to women when she found out about her teammate’s romantic relationship. Joy considered the beginning an “attempt” and an “imitation.” There were lots of lesbians on Joy’s team, and she was trying to fit in with the team:

“I was very out of touch because I was an introvert, but then I dated a great player on the team, and that changed a lot after that; her friends became my friends, and that feeling of having a team made me feel like it was the right thing to try” (Joy).

Nicole started dating girls before she joined the team, and it cannot be said that the sports context influenced her sexuality. Still, Nicole said that the people she dated later were basically teammates. Nicole believes, “it was a natural occurrence when dating a teammate on a women’s team […] my boyish appearance is popular in the team” (Nicole).

In addition, participants believed they spent much time with their teammates, such as training and competing. This intimacy can easily progress into a romantic relationship. “I love volleyball very much, and it’s great to have a close lover who can enjoy volleyball with you” (Zoe).

“I think there are a lot of charming moments (of teammates) on the court. My girlfriend (teammate) and I have some heartfelt feelings that have built up over many moments on the court […] also, we spend more time together with each other” (Kingsley).

Thus, according to participants’ descriptions, the sports context provided a safe environment for female student-athletes to express their sexual orientation. At the same time, it was easy for female student-athletes to develop emotions beyond teammate feelings before a long exposure. Participants see love with their teammates as a bond representing a team climate of intimacy and unity.

### Relative inclusion

When asked how the sports context was for the participants, all participants felt that the sports context was relatively inclusive. This inclusion was firstly reflected in the attitude toward open lesbians from teammates. For example, “the lesbians were free to express their sexual identity here” (Joy). Moreover, Joy stated, “some were just straightforward, ‘this is my girlfriend,” or yes, I like girls’” (Joy). Nicole also provided insight into this point. “It’s different from society; it’s a place for women to express themselves, whether they are heterosexual or homosexual, it does not matter; what matters is that they can express themselves and be accepted at the same time” (Nicole). In addition, “The younger generation is receptive (for sexual minority females)” (Zoe), “[…] in college and on the team, is receptive (for lesbians)” (Kinsley).

Notably, the inclusion attitude also came from the coach. Nicole thought her coach accepted the romantic relationship between her girlfriend and her:

“We have a perfect relationship, and I'm like, like the assistant coach. During the competition season, I will help the coach with much work. I think she trusts me […] she is aware of my girlfriend and will take the initiative to ask about our relationship, ‘how are you doing with her’ […] Yes, she didn't use girlfriend directly. Still, I knew the coach cared about me” (Nicole).

Another aspect of relative inclusion was that participants reported greater acceptance of lesbian and bisexual women than gay men in a sports context. Kinsley stated, “Everyone is acceptable of sexual minority women, right, but the attitude toward gay would be bad” (Kinsley). Zoe explained the different attitudes toward sexual minority women and gay men in the sports context:

“[…] they find that gay to be very scary, and they may find it disgusting. Some guys feel that men can't behave like this. So it is that they will feel disgusted with their demeanor […] It seems that lesbians are fresher now so that the acceptance may be higher” (Zoe).

Thus, relative inclusion refers to the fact that sports contexts are more inclusive of bisexuality than social contexts. In addition, relative to gay men, participants perceived female sexual minorities to be more inclusive.

### Perceived rejection

Although participants reported a relatively positive, inclusive climate in the sports context, prejudice and discrimination still existed:

“I feel like bisexuality is rather less accepted than homosexuality. If you say you only like the same sex, for example, then people will characterize you as gay. But if you're bisexual, you'll be dating women and men, and people might think what you are doing, and it's not acceptable to behave like that” (Zoe).

Joy described her terrible experience with her teammates:

“[…] some of my teammates didn't talk to me anymore, and sometimes I walked into the locker room, and I felt like they were talking about me. It made me feel like a bad person, and I didn't want to face my teammates or myself […] I left (team) for a while” (Joy).

Joy knew her breakup with her teammate was the main trigger, but dating a man exacerbated her teammate’s animosity. “They did not like me that much from the beginning, and this was the opportunity for them to break that silence” (Joy). Similarly, Zoe described being reprimanded by her coach for dating a teammate: “She (coach) told me that she would not allow this on her team and that we both had to be separated or one of us would have to quit the team” (Zoe). In addition, Zoe thought that man (in sports) was more unaccepting of bisexuality: “My ex-boyfriend (male student-athlete) was devastated when he found out that I liked girls, and then he cursed me on social media platforms” (Zoe).

Although other participants reported not experiencing direct conflict, they would hear some bad comments about being homosexual or bisexual. “You know, sometimes people will imitate or laugh at some lesbian or gay gestures. It’s common […] and as long as it’s not against me, that’s fine” (Nicole). “My parents think it’s a disease, and I have no way to communicate with them” (Kinsley).

Participants experienced direct and indirect rejection regarding bisexual identity. These rejections come from their teammates, coaches, close people, and parents, and these are often the people they want to get support from.

## Discussion

This study explored the experiences of female bisexual student-athlete in China, particularly focused on examining the perceptions and experiences of female bisexual student-athletes regarding bisexual identity in the sports context.

The results show that bisexual identity is invalid in the sports context, which is consistent with previous findings in other contexts. Researchers found that bisexual individuals had more frequent experiences of perceived identity denial and higher meta-illegitimacy (Garr-Schultz and Gardner, 2021; Maimon et al., 2021; Thöni et al., 2022). Experience of bisexual denial often comes from endorsing bisexual stereotypes by others and the perception that bisexuality is an illegitimate identity (Maimon et al., 2021). In this study, bisexual identity was considered as lesbian, not a real sexual orientation, an unimportant thing; those are evidence of bisexual denial. As a response, female bisexual student-athletes perceived their identities as confusing and wrong, creating an internalized bisexual denial. The stress process of female bisexual student-athletes is consistent with minority stress theory (Meyer, 2003). In addition, self-acceptance of one’s sexual orientation is an essential aspect of mental health for bisexual individuals (Camp et al., 2020), and experiencing identity denial was associated with depressive symptoms, low self-esteem, and low well-being (Garr-Schultz and Gardner, 2021; Maimon et al., 2021; Thöni et al., 2022). Thus, for some participants, the bisexual denial experiences and self-doubt posed severe mental health problems. Although Flanders et al. (2019) found that social support can reduce anxiety and depression in bisexual youth, the participants did not receive the support they needed, according to the findings established in this study.

The findings also emphasize the importance of including emotion as an integral part of the definition of bisexuality. Swan (2018) indicated that previous studies have often overlooked emotional factors. Omitting emotion when measuring sexual orientation creates research bias because emotion is more strongly correlated with self-identity than behavior. Perrotta (2020) summarized relevant scientific research indicating that in analyses of the whole brain, bisexual women tend to show more mixed activation patterns: areas associated with sensory processing domains that are more sensitive to female stimuli; In contrast, areas associated with social cognition are more sensitive to male stimuli. This could explain our findings that in women’s teams, female bisexual student-athletes emphasize sensory attraction, while in society, they tend to be heterosexual. Additionally, Swan (2018) noted that measuring the effect of emotion can be tricky, while our findings revealed that security was the emotional component of concern for female bisexual student-athletes. This finding could help researchers target the emotional component of the definition of bisexuality.

Diamond (2008) proposed the concept of “situation-dependent flexibility in women’s responsiveness” to explain women’s sexual fluidity; this study also found similar phenomena in the experiences of female bisexual student-athletes. However, unlike Diamond’s focus on love and desire in sexual fluidity, the results of this study suggest that participants’ sexual fluidity was not only driven by intimate emotions but also influenced by social pressures. Bisexual females were attracted to women because of intimate emotions, while their sexuality turned to men due to family and social pressures; as one participant said, “choose the easy way.” Therefore, more research needs to consider the impact of family and social pressures on the sexual fluidity of bisexual women.

Despite the belief that same-sex couples should be more equal, Doan and Quadlin (2019) have found that heterosexual norms were adopted between same-sex couples: butch and femme would take on different domestic responsibilities that society expects of men and women. Similarly, in this study, female bisexual athletes must choose and play male or female roles. Additionally, as a similar term to “T,” butch is often mentioned in lesbian-related studies in sports and used in an unfriendly way (Waldron, 2016). However, according to the participants’ descriptions in this study, both lesbian and bisexual student-athletes accepted the term. This may be due to the prevalence of the heterosexual culture in China. Wang (2021). Found that under the influence of China’s dominant heteronormative culture and globalization, lesbian culture in China had developed a hierarchy. One hierarchy was between masculinity and femininity among lesbians, which encourages various practices of female masculinity but still values masculinity over femininity.

The influence of the sports context on female sexual fluidity can be explained by two aspects. On the one hand, this may be explained by the level of intimacy that is socially acceptable for women. Rodríguez Rust (2000) identified that western culture allows women to express emotions and be more intimate in relationships with other women and men than men. Thus, among women, sexual intimacy may be an outgrowth of socially acceptable emotional intimacy; this may contribute to greater flexibility by giving women greater freedom than men to explore their deep feelings for members of the same sex. As in this study, prolonged exposure to sports training and competition makes it easy for female student-athletes to be better able to explore intimate relationships. On the other hand, the influence may be linked to the women’s life history strategies (Luoto et al., 2019a,b; Luoto and Rantala, 2020). Life history strategies assume that heterosexual males have faster life history strategies than heterosexual females, such as focusing on mating, risky behaviors, etc. To the extent that non-heterosexual women exhibit physical, psychological, and behavioral masculinities, their life history strategies and outcomes also exhibit more typical masculinities. The participants in this study were all in competitive sports, requiring more robust sports performance. Thus, sports requirements may influence sexual fluidity by influencing the life history strategies of female student-athletes, as fast life history strategies for non-heterosexual identities are better adapted to the needs of the sports context. Additionally, Luoto and Rantala (2020) indicated that bisexual women have more male-typical personality traits, which could support our hypothesis.

Moreover, the finding of a relatively inclusive climate in women’s teams is consistent with previous studies. For example, in Mann and Krane (2018)’s study, 13 female queer student-athletes (three bisexuals) reported on the inclusive climate and transition inclusive climate in the collegiate sports context. Similarly, Pariera et al. (2021) also found that for concerns about being an LGBTQ athlete, women were less likely to worry about being alienated from their team. However, it is worth noting that these positive findings may come from high athletic capital (Anderson and Bullingham, 2015; Halbrook et al., 2019). That means athletes with high sports capital in the team will get more inclusion. This situation is similar in our study, as two of the participants had been team captains, and two others were also good athletes. Hence, this relatively inclusive climate of female bisexual student-athletes in China may require further research.

Furthermore, participants described some negative experiences, but not all these negative experiences came from bisexual identity. Similar yet subtle differences exist in the negative experiences of bisexuals and lesbians in the sports context, which will need to be investigated in depth in future studies. However, social non-acceptance and the lack of sexual knowledge among citizens are common challenges for sexual minorities in China. One national survey found that Chinese youth have low sexual knowledge (Lyu et al., 2020). Inadequate sex education may lead to a lack of diversity and inclusion in the sexual orientation of Chinese youth. Therefore, increasing sexual knowledge is a prerequisite for improving bisexuality-related issues. Sex education is urgently needed in China to address the social problems related to sexuality, for example, to incorporate LGBTQ-related knowledge into sex education in China (Chi et al., 2015).

## Implications

This study extends the limited research on bisexuality-related research in sports by exploring the experiences of female bisexual student-athletes in China. It provides an intersectionality study on the development of bisexual identity. Understanding the development of a global perspective on bisexuality requires a careful understanding of each cultural context. Because the experiences of LGBTQ individuals vary widely over time and geography (Anderson and Piedra, 2021). The exploration of bisexual identity and its relationship with heterosexual norms in this study gives a different cultural context and practical basis for bisexuality-related research. Given the experience of bisexuality in research as distinct from lesbianism, we emphasize the importance of the need to study bisexuality as a distinct identity.

To create a more inclusive climate for female bisexual student-athletes, the government, higher education, and sports organizations can utilize the adoption of anti-discrimination and inclusion policies. Our findings provide evidence to support that these policies should include special attention to sex education issues and the inclusion of sexual orientation diversity. Coaches and non-bisexual student-athletes can also benefit from the study. Coaches and non-bisexual student-athletes can use the study to understand the experiences of their bisexual teammates better and pay attention to behaviors that promote higher levels of team cohesion and performance.

The theoretical perspective of the study was the minority stress theory (Meyer, 2003). Minority stress theory is an applicable theory in the study of sexual minorities. This study extended the minority stress theory by interpreting the experiences of female bisexual student-athletes in China.

## Limitation

Although this study yielded important information about the unique experiences of female bisexual student-athletes in China, like all studies, it is not without limitations. Our findings do not represent the broader population of female bisexual student-athletes, as our sample was limited to a specific geographic setting in eastern China. Therefore, it cannot be assumed that the experiences and perceptions of the participants are the same as those of other female bisexual student-athletes. In addition, participants who agreed to be interviewed may differ in characteristics or experiences from those who did not agree to be interviewed. For example, female bisexual student-athletes with more positive sports experiences may have been more willing to participate in the study. At last, our participants were all graduate students. Although both undergraduate and graduate students compete together in China, the experiences of undergraduate student-athletes may differ from those of graduate students.

## Conclusion

Even today, sexual minority women in many cultural contexts are subject to various forms of prejudice and discrimination. At the same time, the heterosexist system of sports has created resistance to sexual minority women’s sports participation. We should work harder to make the sports context more equal and inclusive. This study provides new insight into bisexuality-related research by exploring the experiences of female bisexual student-athletes in the Chinese sports context. Although this is an initial exploration, we hope to advance research on female sexual minorities in the Chinese context and other intersectional studies on sexual identity-related issues.

Future research needs to explore the relationship between bisexual identity formation and the sports context in the Chinese context. Meanwhile, research is required to identify additional reasons for the overlap of bisexual and lesbian identities in the sports context. Also, quantitative research is recommended to investigate attitudes toward bisexuality in sports contexts, providing a context for bisexuality-related research in sports. At the same time, more attention needs to be paid to cultural contexts in studies in Asian countries to expand on issues related to bisexual sports participation in a different cultural setting.

## Data availability statement

The raw data supporting the conclusions of this article will be made available by the authors, without undue reservation.

## Ethics statement

The studies involving human participants were reviewed and approved by the Ethics Committee for Research involving Human Subjects Universiti Putra Malaysia. The patients/participants provided their written informed consent to participate in this study.

## Author contributions

MX: conceptualization, methodology, data curation, and writing—original draft preparation. MX and YX: data collection and data analysis. KS, SA, and NZ: writing—review and editing and supervision. All authors have read and agreed to the published version of the manuscript.

## Conflict of interest

The authors declare that the research was conducted in the absence of any commercial or financial relationships that could be construed as a potential conflict of interest.

## Publisher’s note

All claims expressed in this article are solely those of the authors and do not necessarily represent those of their affiliated organizations, or those of the publisher, the editors and the reviewers. Any product that may be evaluated in this article, or claim that may be made by its manufacturer, is not guaranteed or endorsed by the publisher.
