# Supplementary material for: The experiences of female bisexual student-athletes in China: An interpretative phenomenological analysis
Source: Front Psychol. 2023 Mar 22;14:1129961. doi: 10.3389/fpsyg.2023.1129961 (PMC10074485; doi:10.3389/fpsyg.2023.1129961)
Supplement: Supplementary file 1 [file Data_Sheet_1.docx]

**Appendix 1. Interview Guide.**

| **Interview Guide** |
| --- |
| 1. Could you describe when you started having thoughts consistent with sexual orientation? 2. Could you tell us what your perception of bisexuality is? 3. Can you reflect on what the bisexual identity means for you? 4. How do/did you feel about your bisexual identity in the sports context? 5. How do you perceive the impact of the sports context on the sexual orientation of female student-athletes? 6. Can you tell me the differences and similarities between bisexual females and lesbians in the sports context? 7. How do you think your teammates and coaches perceive you as a bisexual student-athletes? 8. Have you experienced any negative experiences regarding bisexual identity in the sports context?   *Possible prompts:*  *(1) When did it happen?*  *(2) How did you feel?*  *(3) How did you react?*   1. Did you witness or hear any negative experiences of bisexual individuals in the sports context?   *Possible prompts:*  *(1) When did it happen?*  *(2) How did you feel?*   1. Did you disclose your bisexual identity to your coaches and teammates?   *If the participant did, the following questions would be asked:*  *(1). When did you disclose your bisexual identity to them?*  *(2). How did you disclose your bisexual identity to them?*  *(3). Why did you disclose your bisexual identity to them?*  *If the participant did not, the following questions would be asked:*  *(d). Why did you close your bisexual identity to them?*   1. How would you feel if the team rejected you because of your sexual orientation? |
